# Supplementary figures and images for: Proper control of R‐loop homeostasis is required for maintenance of gene expression and neuronal function during aging
Source: Aging Cell. 2022 Jan 20;21(2):e13554. doi: 10.1111/acel.13554 (PMC8844117; doi:10.1111/acel.13554)

**A**Rh1>GFP<sup>KASH</sup>Rh1>GFP<sup>KASH</sup>

Rh1&gt;siControl

D10

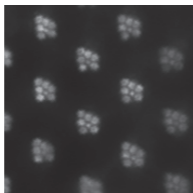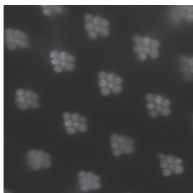

D20

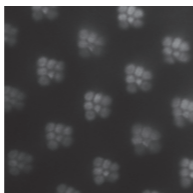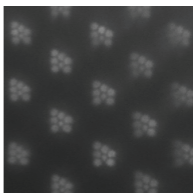

D30

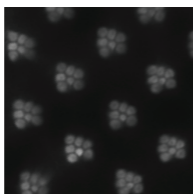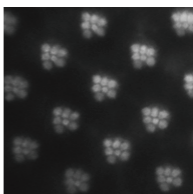

D40

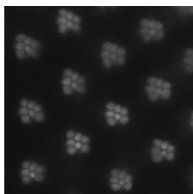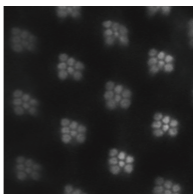

D50

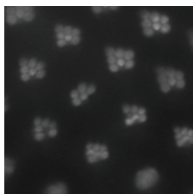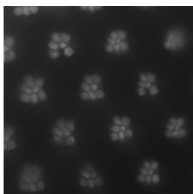

Supplement: Supplementary file 4 — Figure S1 [file ACEL-21-e13554-s002.pdf]

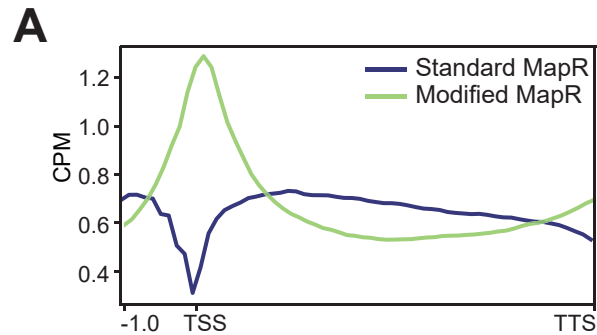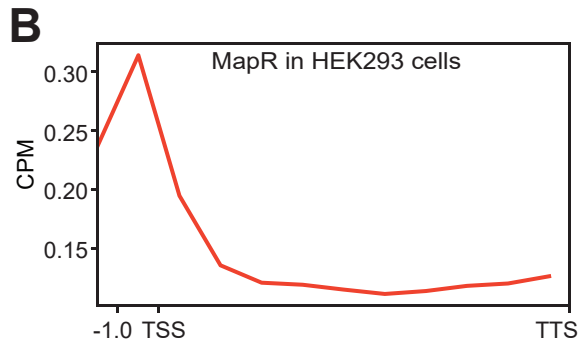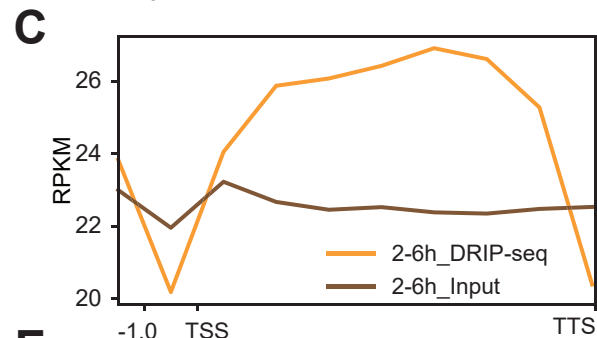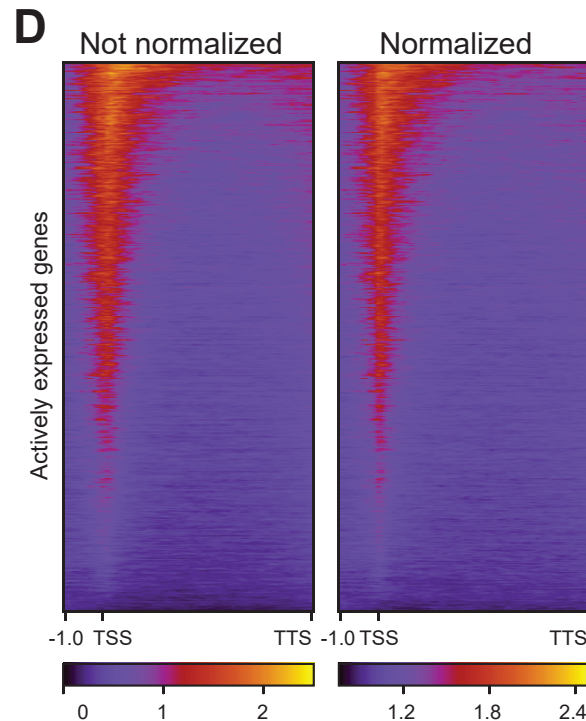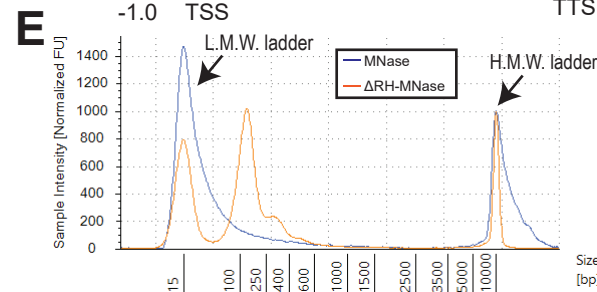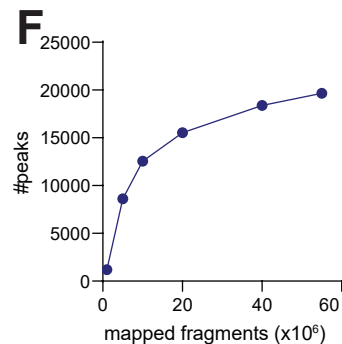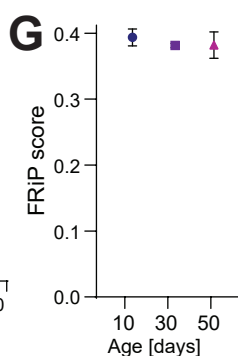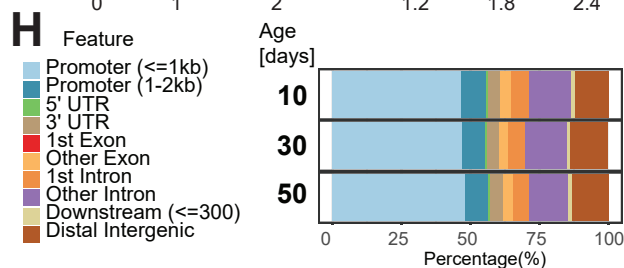

Supplement: Supplementary file 5 — Figure S2 [file ACEL-21-e13554-s006.pdf]

**A**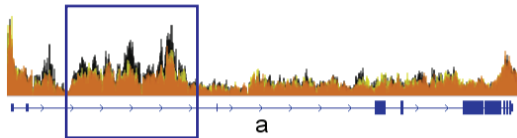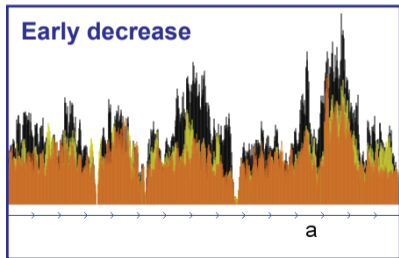

Age  
D10  
D30  
D50

**Late increase**

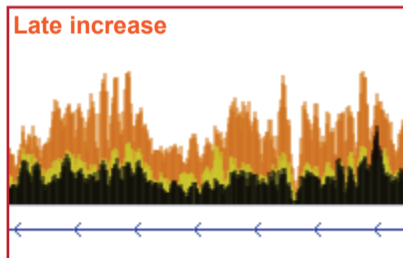**B**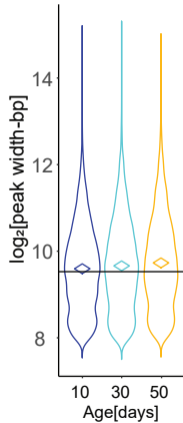

Supplement: Supplementary file 6 — Figure S3 [file ACEL-21-e13554-s005.pdf]

**A**

| M.S. (n=4) |           |            |
|------------|-----------|------------|
| Age        | Abundance | Normalized |
| 10         | 120.6     | 1.01       |
| 10         | 113.3     | 0.95       |
| 10         | 126.5     | 1.06       |
| 10         | 116.5     | 0.98       |
| 40         | 105.7     | 0.89       |
| 40         | 99.3      | 0.83       |
| 40         | 91.2      | 0.76       |
| 40         | 94        | 0.79       |

**B**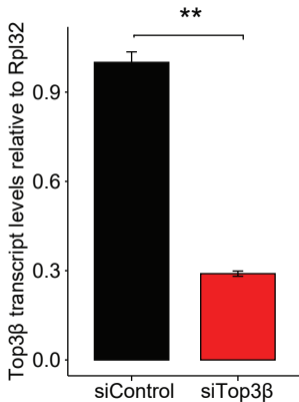

Supplement: Supplementary file 7 — Figure S4 [file ACEL-21-e13554-s004.pdf]
