# Supplementary material for: Proper control of R‐loop homeostasis is required for maintenance of gene expression and neuronal function during aging
Source: Aging Cell. 2022 Jan 20;21(2):e13554. doi: 10.1111/acel.13554 (PMC8844117; doi:10.1111/acel.13554)
Supplement: Supplementary file 8 — Figure S5 [file ACEL-21-e13554-s007.pdf]

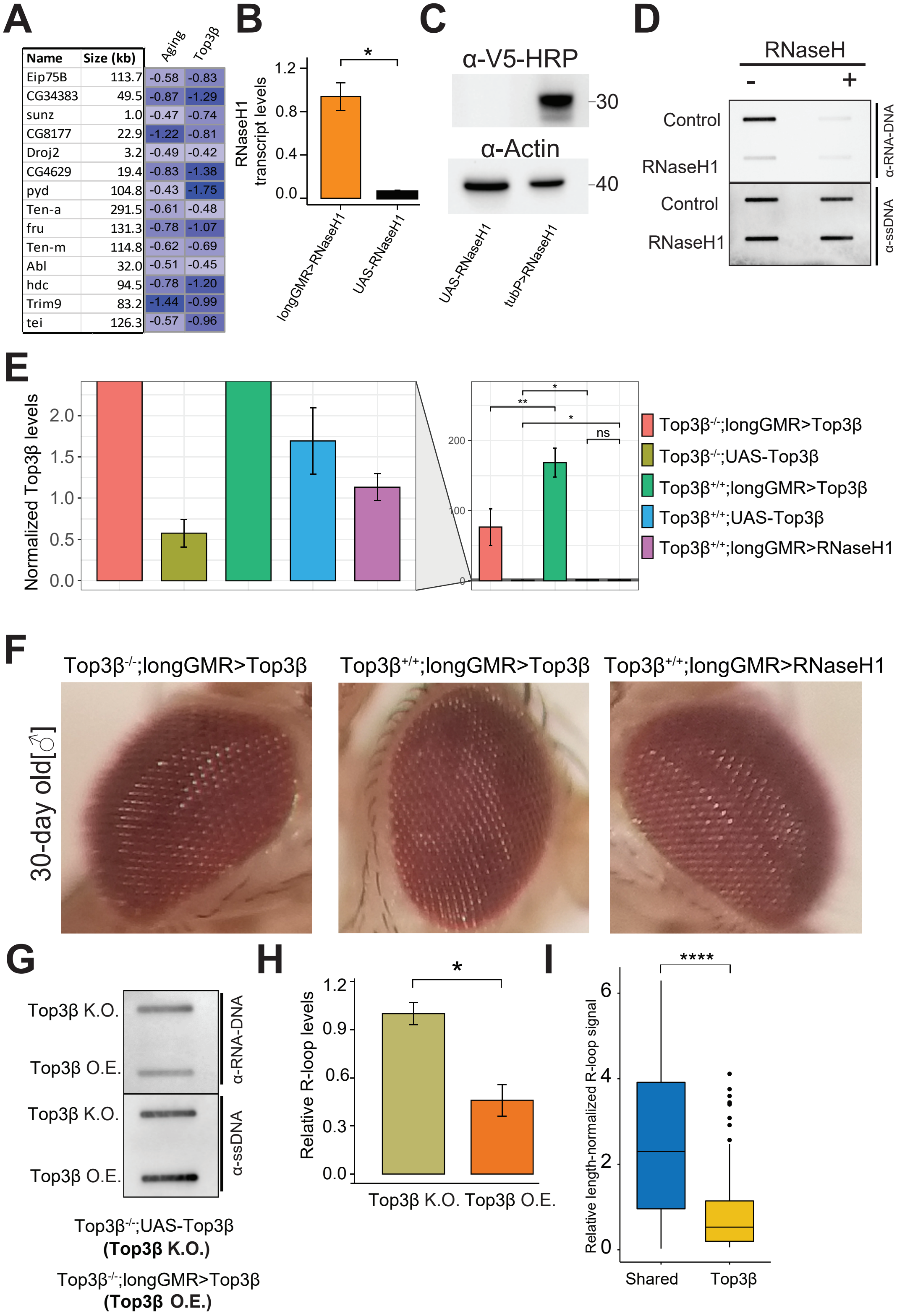
A
Aging
Top3β
Name Size (kb)
Eip75B 113.7 -0.58 -0.83
CG34383 49.5 -0.87 -1.29
sunz 1.0 -0.47 -0.74
CG8177 22.9 -1.22 -0.81
Droj2 3.2 -0.49 -0.42
CG4629 19.4 -0.83 -1.38
pyd 104.8 -0.43 -1.75
Ten-a 291.5 -0.61 -0.48
fru 131.3 -0.78 -1.07
Ten-m 114.8 -0.62 -0.69
Abl 32.0 -0.51 -0.45
hdc 94.5 -0.78 -1.20
Trim9 83.2 -1.44 -0.99
tei 126.3 -0.57 -0.96
B
RNaseH1 transcript levels
1.2
0.8
0.4
0.0
*
longGMR>RNaseH1
UAS-RNaseH1
C
α-V5-HRP
-30
α-Actin
-40
UAS-RNaseH1
tubP>RNaseH1
D
RNaseH
-
+
Control
RNaseH1
Control
RNaseH1
α-RNA-DNA
α-ssDNA
E
Normalized Top3β levels
2.0
1.5
1.0
0.5
0.0
200
100
0
*
**
*
ns
Top3β-/-;longGMR>Top3β
Top3β-/-;UAS-Top3β
Top3β+/+;longGMR>Top3β
Top3β+/+;UAS-Top3β
Top3β+/+;longGMR>RNaseH1
F
Top3β-/-;longGMR>Top3β
Top3β+/+;longGMR>Top3β
Top3β+/+;longGMR>RNaseH1
30-day old[♂]
G
Top3β K.O.
Top3β O.E.
Top3β K.O.
Top3β O.E.
α-RNA-DNA
α-ssDNA
Top3β-/-;UAS-Top3β
(Top3β K.O.)
Top3β-/-;longGMR>Top3β
(Top3β O.E.)
H
Relative R-loop levels
1.2
0.9
0.6
0.3
0.0
*
Top3β K.O.
Top3β O.E.
I
Relative length-normalized R-loop signal
6
4
2
0
****
Shared
Top3β
